# Unusual Presentation of Pemphigus Vulgaris as a Solitary Ulcerative Scalp Lesion: A Case Report

**DOI:** 10.7759/cureus.87025

**Published:** 2025-06-30

**Authors:** Sara Nejjari, Ghita Basri, Inas Chikhaoui, Khalqui Slamti, Soumiya Chiheb

**Affiliations:** 1 Dermatology, Cheikh Khalifa International University Hospital, Mohammed VI University of Health Sciences, Casablanca, MAR

**Keywords:** atypical pemphigus vulgaris, autoimmune blistering skin disease, delayed diagnosis, dermoscopy, scalp lesion, solitary ulcer

## Abstract

Pemphigus vulgaris (PV) is a rare autoimmune blistering disease that typically presents with painful oral erosions followed by widespread cutaneous involvement. We report a diagnostically challenging case of PV in a 43-year-old man who initially presented with a solitary, ulcerative-crusted lesion on the parietal scalp, evolving over eight months. The lesion was clinically suggestive of infectious or neoplastic etiologies, including tinea capitis, squamous cell carcinoma, Bowen's disease, and leishmaniasis. Empirical antifungal treatment was ineffective, and mycological studies were negative. Dermoscopy revealed yellow crusts, serpentine vessels, and perifollicular whitish halos. The patient later developed painful oral ulcers, which led to histopathological and immunofluorescence studies confirming PV. Systemic corticosteroids and mycophenolate mofetil were initiated with marked clinical improvement. This case highlights an uncommon presentation of PV that delayed diagnosis and emphasizes the importance of considering autoimmune bullous disorders in the differential diagnosis of chronic, non-healing scalp lesions, even in the absence of mucosal involvement.

## Introduction

Pemphigus vulgaris (PV) is a rare autoimmune blistering disorder primarily affecting middle-aged adults with an estimated annual incidence of 0.1-0.5 per 100,000 individuals worldwide. It is characterized by intraepidermal acantholysis and the formation of flaccid blisters and erosions on the skin and mucous membranes, mediated by autoantibodies against desmoglein 3 (Dsg3) and, in mucocutaneous cases, desmoglein 1 (Dsg1). The disease typically begins with painful erosions of the oral mucosa, later progressing to widespread cutaneous involvement. However, atypical and localized presentations can occur, which may significantly delay diagnosis and appropriate treatment [1,2].

Scalp involvement is well documented in pemphigus but rarely constitutes the initial or sole manifestation of the disease [3]. In such cases, PV may mimic other conditions like pemphigus foliaceus (PF), which primarily targets Dsg1 and typically presents with superficial erosions and crusting, sparing the mucous membranes. Unlike PV, PF lesions are often localized to the seborrheic areas, including the scalp, face, and upper trunk, and histopathology reveals subcorneal acantholysis. The differential diagnosis between PV and PF is crucial, as their management and prognosis differ. Additionally, other proteins such as plakins and desmocollins may play a role in the pathophysiology of scalp lesions, further complicating the clinical picture [4].

Among these atypical presentations, scalp involvement has been identified as a significant yet often under-recognized site. According to Sar-Pomian et al., the scalp may be involved in various subtypes of pemphigus, including PV and PF, with lesions sometimes appearing as the first or sole manifestation [3].

This report describes an unusual case of PV in a 43-year-old man presenting initially with a single ulcerated and crusted lesion on the parietal scalp, clinically mimicking infectious or neoplastic conditions, leading to a delay in diagnosis. We also review similar cases and dermoscopic features reported in the literature.

## Case presentation

A 43-year-old man with no significant past medical history presented to the dermatology clinic with a single, non-healing lesion on the parietal region of the scalp. The lesion had been evolving progressively over the past eight months, with gradual enlargement, pruritus, and localized tenderness. The patient denied any fever, systemic symptoms, or mucosal involvement at the initial presentation.

**Figure 1:**
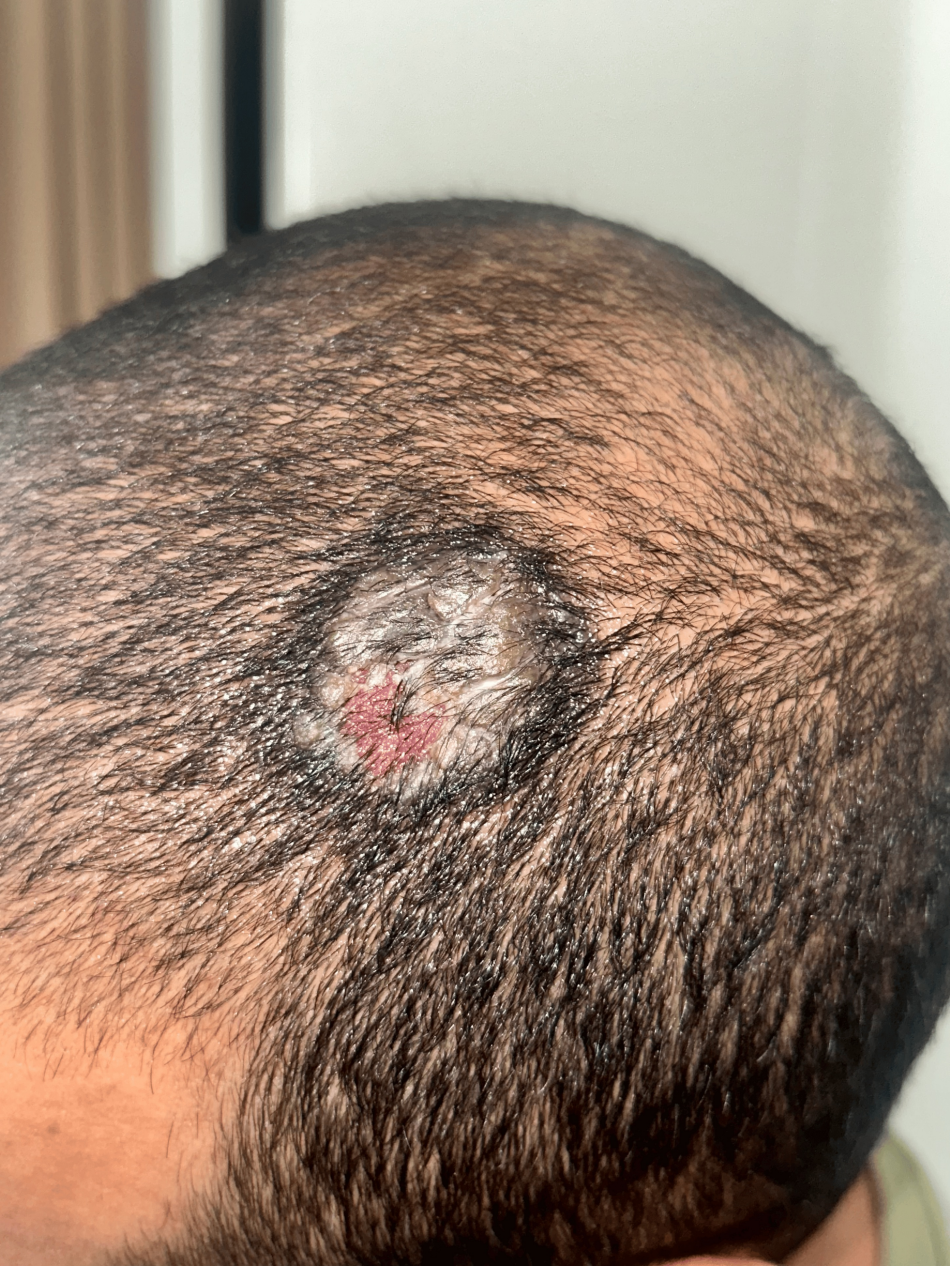
Solitary, well-demarcated, ulcerative plaque measuring approximately 4 cm in diameter of the scalp

On physical examination, the lesion was observed as a solitary, well-demarcated ulcerative plaque measuring approximately 4 cm in diameter (Figure 1). The surface was covered with yellowish crusts and revealed a moist, erythematous base underneath. The border of the lesion appeared infiltrated and slightly elevated, while the plaque itself was indurated upon palpation and tended to bleed with minimal trauma. There was no evidence of regional lymphadenopathy or additional cutaneous involvement at that time.

Given the clinical presentation, several differential diagnoses were considered, including tinea capitis (kerion form), cutaneous leishmaniasis, Bowen's disease, squamous cell carcinoma, and chronic bacterial or fungal infections. The patient was initially treated empirically with oral griseofulvin at a dose of 500 mg/day for six weeks, but no clinical improvement was observed. Subsequent mycological examination and culture of skin scrapings were negative for fungal elements, and bacterial swabs were also sterile, effectively ruling out infectious etiologies.

**Figure 2:**
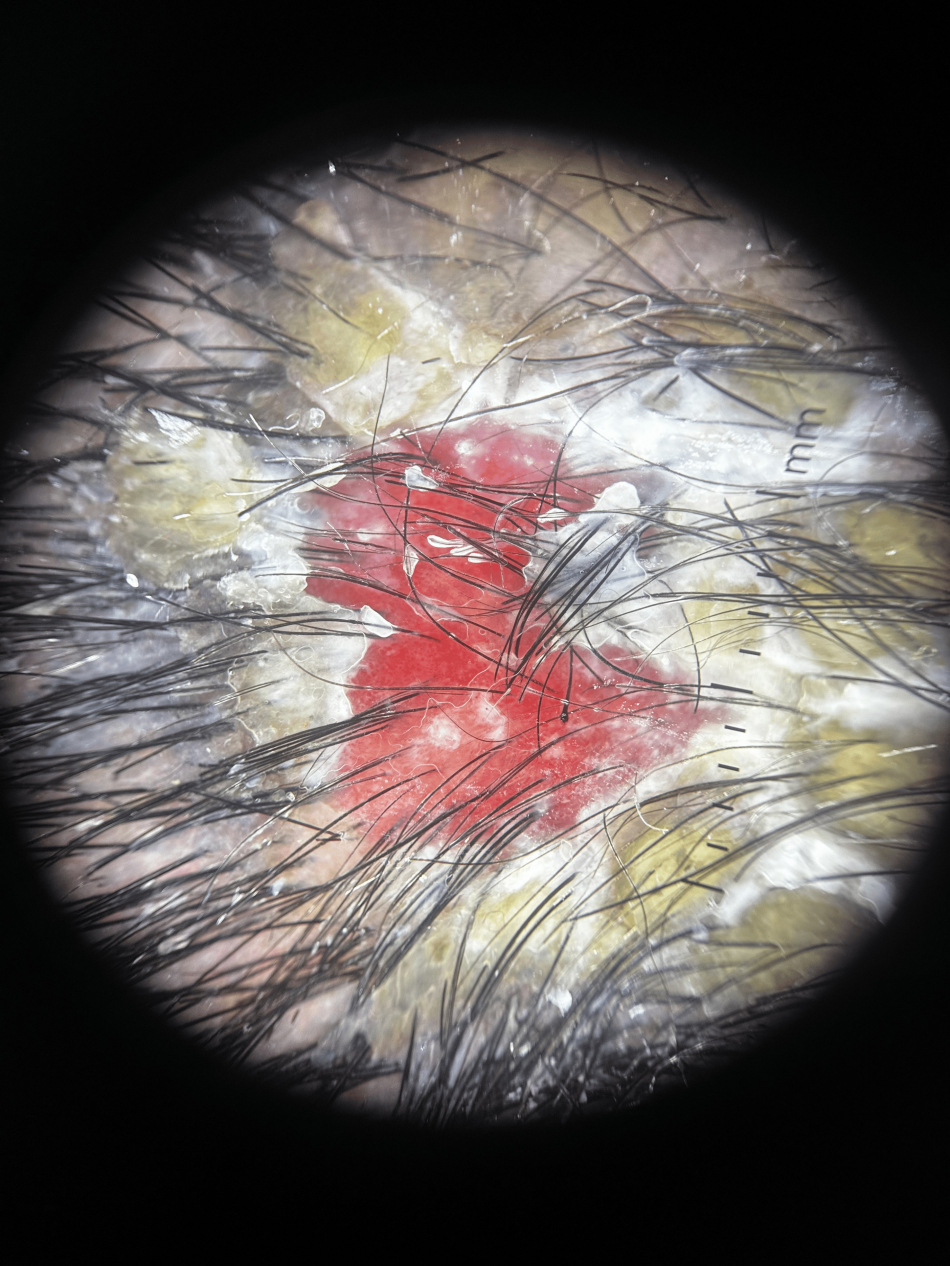
Dermoscopy image (DermLite DL5) showing yellowish crusts on an erosive base, irregular linear vessels, and scattered hemorrhagic dots

To further characterize the lesion, dermoscopy was performed using a DermLite DL5 dermatoscope (Aliso Viejo, California, United States). The examination revealed yellowish crusts over an erosive base, along with serpentine and irregular linear vessels and multiple hemorrhagic dots. Notably, white halos were seen surrounding follicular openings, producing what is referred to as the "fried egg sign" (Figure 2). These dermoscopic features suggested an inflammatory, possibly autoimmune origin rather than an infectious or neoplastic process.

**Figure 3:**
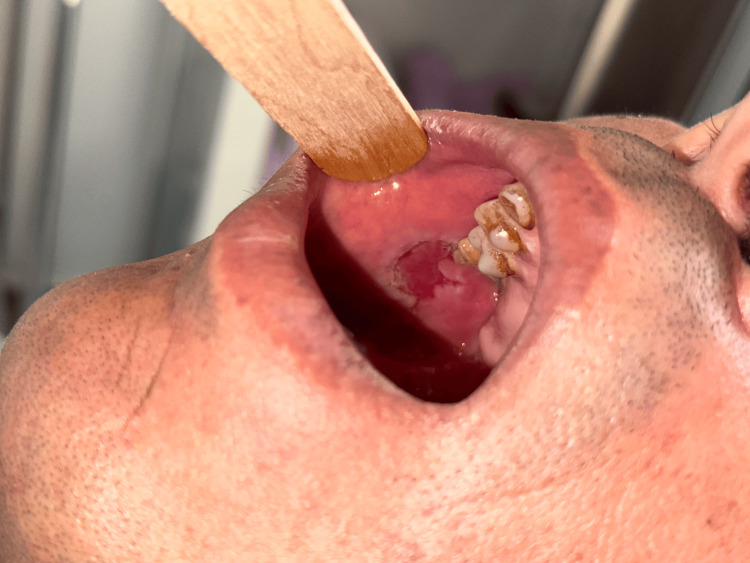
Erosion and ulcer on the buccal mucosa

Two months following the initial consultation, the patient reported the sudden onset of severe oral pain and difficulty swallowing. Clinical examination at that stage revealed multiple erosions and ulcers involving the buccal mucosa, soft palate, and inner aspects of the lips (Figure 3). In light of the progression and new mucosal involvement, two punch biopsies were obtained from the scalp: one from an active lesional area for routine histopathological examination and another from adjacent perilesional skin for direct immunofluorescence.

The severity of the disease was assessed using the Pemphigus Disease Area Index (PDAI), which separately evaluates skin, scalp, and mucosal involvement. At initial presentation, the PDAI score was 5 for the skin and 4 for the scalp, reflecting a single, moderately sized localized lesion without mucosal involvement (mucosal score: 0). Two months later, following the appearance of mucosal lesions, the mucosal score was 8, raising the total PDAI to 17. This standardized assessment highlights the clinical progression from isolated scalp involvement to more extensive disease with mucosal involvement, thereby facilitating the objective monitoring of disease progression and treatment response.

**Figure 4:**
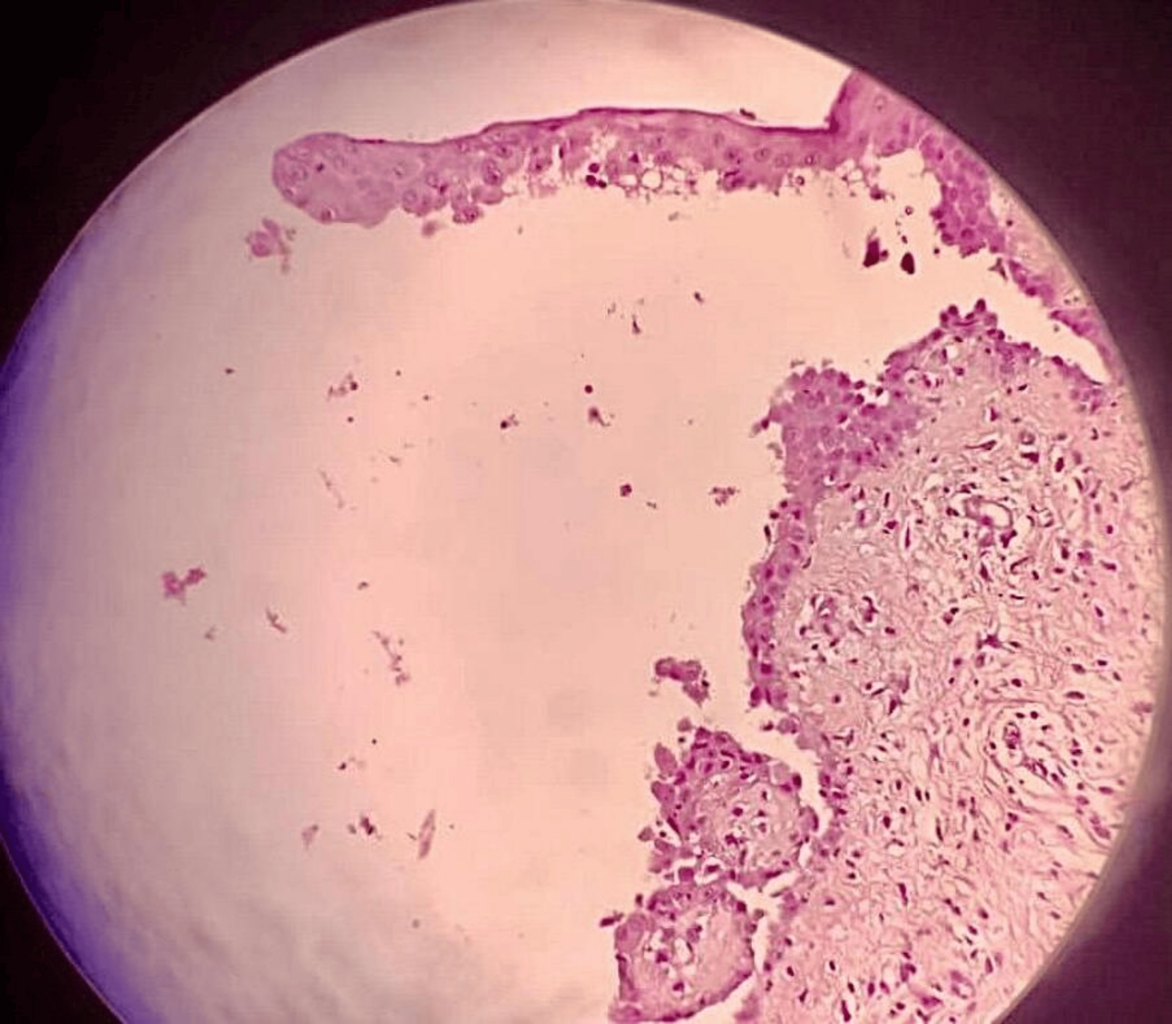
Acantholysis with "row of tombstone" appearance of basal keratinocytes

**Figure 5:**
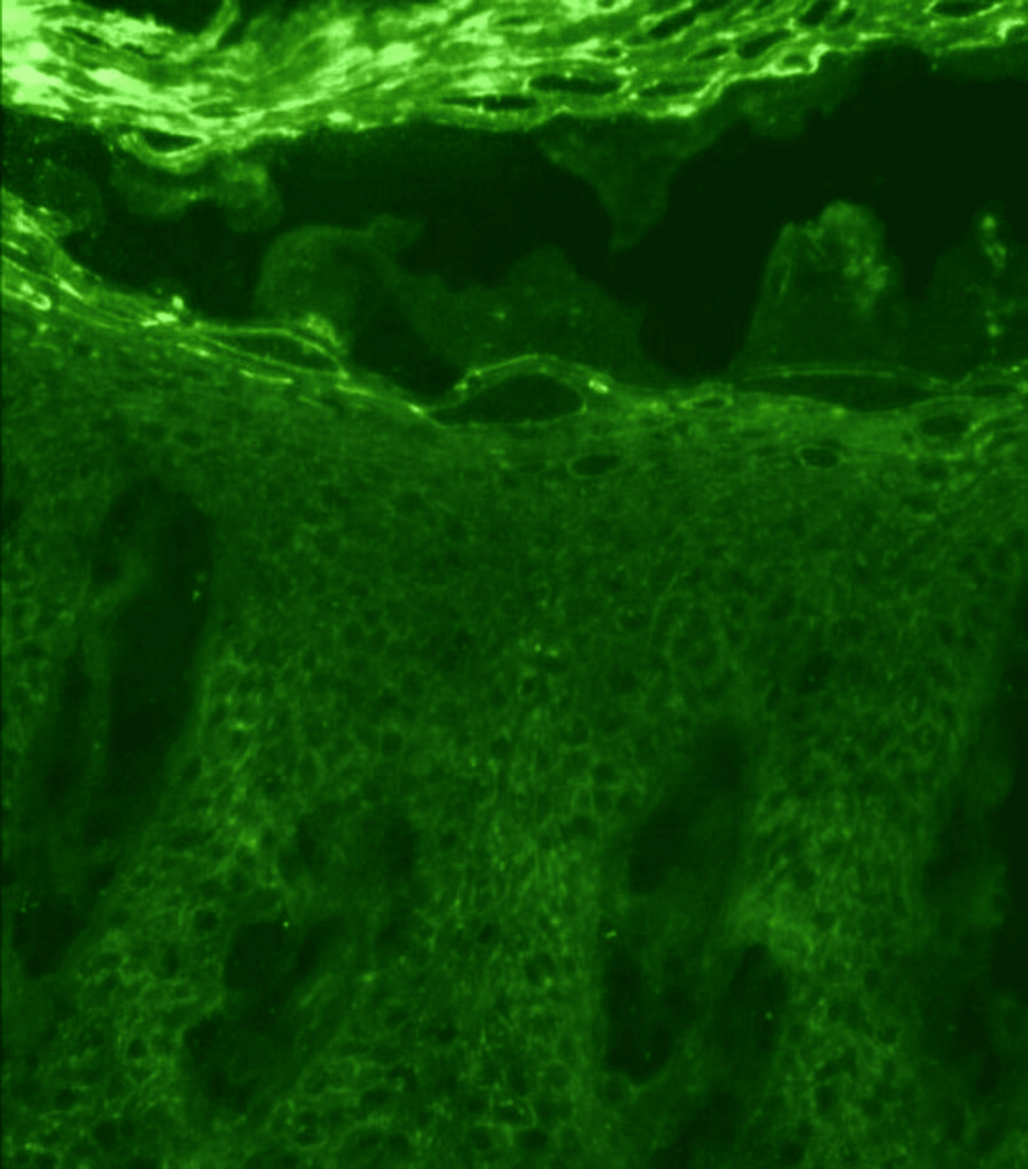
Intercellular IgG and C3 deposits in a net-like pattern within the epidermis

A punch biopsy sample was obtained from an active area of the lesion, including both lesional and perilesional skin. Routine histopathological examination of the lesional tissue demonstrated suprabasal acantholysis with the classic "row of tombstone" appearance, reflecting the detachment of suprabasal keratinocytes, while basal cells remained attached to the basement membrane (Figure 4). To confirm the suspected diagnosis, a perilesional skin biopsy was sent for direct immunofluorescence. The sample was transported in Michel's medium and processed using fluorescein-labeled antibodies. Direct immunofluorescence analysis revealed intercellular deposits of IgG and complement component C3 in a net-like (reticular) pattern throughout the epidermis, which is diagnostic of PV (Figure 5).

Following confirmation of the diagnosis, prior to initiating systemic immunosuppressive therapy, a comprehensive baseline safety assessment was conducted. This included a complete blood count, liver function tests, renal function tests, and serologic screening for hepatitis B, hepatitis C, and human immunodeficiency virus (HIV). All laboratory values were within normal limits, and no contraindications to immunosuppressive treatment were identified. The patient was started on systemic corticosteroid therapy with oral prednisone at 1 mg/kg/day, in combination with mycophenolate mofetil at a dose of 2 g/day. This therapeutic approach led to a rapid resolution of the oral mucosal lesions and progressive re-epithelialization of the scalp ulcer over the following weeks.

## Discussion

PV typically begins with painful oral erosions, followed by skin involvement, with scalp lesions generally occurring later in the disease [1,2]. However, the atypical presentation in this case, with a solitary ulcerative lesion on the scalp, posed a significant diagnostic challenge. Initially, the lesion was suspected to be tinea capitis, squamous cell carcinoma, Bowen's disease, or cutaneous leishmaniasis, all common diagnoses for ulcerative lesions on the scalp [5-8]. This was further complicated by the absence of typical PV signs, such as oral erosions or widespread blisters [9].

To contextualize our case within the existing literature, Table 1 summarizes previously reported cases of pemphigus presenting with scalp involvement. These cases vary in patient demographics, lesion location, clinical features, diagnostic methods, and treatment approaches. Our patient's presentation is distinguished by isolated involvement of the parietal scalp with a delayed onset of mucosal lesions, which is rare compared to other cases reporting simultaneous or early mucosal involvement. This comparison underscores the diagnostic challenges and highlights the importance of considering pemphigus even in atypical localized scalp lesions [10-12].

**Table 1:** Comparative analysis of reported cases of pemphigus with scalp involvement DIF: direct immunofluorescence

Study/case	Patient age and sex	Lesion location	Clinical presentation	Diagnostic method	Treatment	Unique features
Altmann et al., 2021 [10]	68, female	Scalp only	Alopecic and scabby plaque	Histopathology + DIF	Topical corticosteroids	Excellent response with hair regrowth
Bosseila et al., 2019 [11]	40, male	Scalp only	Patchy alopecia, erythema, follicular pustules	Histopathology + DIF	Systemic corticosteroids + mycophenolate mofetil	Mimicked folliculitis decalvans
Vega-Diez et al., 2021 [12]	68, female	Scalp only	Alopecic and scabby plaque	Histopathology + DIF	Topical corticosteroids	Excellent response with hair regrowth
Our case	43, male	Isolated parietal scalp	Single non-healing ulcer with delayed mucosal onset	Histopathology + DIF	Prednisone + mycophenolate mofetil	Rare isolated scalp onset, delayed mucosal lesions

The differential diagnosis in this case was complicated by the lesion's appearance, which initially pointed to a fungal infection, particularly tinea capitis, given the crusted nature and localized aspect of the lesion [6,13]. The failure of antifungal treatment and negative mycological tests helped exclude this possibility. The lesion's infiltrated borders and slow progression also suggested a potential malignant process, such as squamous cell carcinoma or Bowen's disease, both of which can present as chronic, non-healing ulcers [7,8,14,15]. However, histopathological examination revealed suprabasal acantholysis and intraepidermal blister formation, characteristic of PV, which led to the correct diagnosis [14,16].

The diagnostic delay was partly due to the isolated nature of the lesion, which is uncommon in PV, where lesions are usually widespread and symmetrical [2,9]. This unusual presentation led to misdiagnosis and ineffective treatment with griseofulvin, followed by extensive investigations without reaching a conclusion. Dermoscopy played a critical role in raising early suspicion for an autoimmune origin. In particular, the observation of the "fried egg sign" consisting of a central yellowish crust surrounded by a white perifollicular halo is a dermoscopic feature described in autoimmune inflammatory scalp conditions, including lichen planopilaris and discoid lupus erythematosus [17]. While this sign is not specific to PV, its presence helped steer the diagnostic orientation away from infectious or neoplastic etiologies. The differential diagnosis highlights the importance of considering autoimmune diseases, such as PV, even when the clinical presentation initially suggests infectious or neoplastic conditions.

Sar-Pomian et al. conducted a comprehensive review of scalp involvement in pemphigus and emphasized that scalp lesions may present as isolated findings, particularly in early stages or less typical variants such as PF. The authors reported that in some cases, these lesions precede mucosal or generalized skin involvement. This reinforces the diagnostic relevance of recognizing unusual scalp presentations and correlates well with our patient's course, where the scalp lesion was the only initial sign of PV [3].

This addition further emphasizes the diagnostic difficulty posed by this atypical presentation of PV and underscores the importance of maintaining a high degree of suspicion for autoimmune blistering diseases, especially in cases that do not fit the typical clinical pattern [18].

## Conclusions

This case report underscores an atypical and rare presentation of PV, manifesting solely as a solitary, ulcerative-crusted lesion on the scalp, without initial mucosal involvement or widespread cutaneous blisters. Such a presentation deviates from the classic clinical profile of PV, where painful oral erosions are typically the first manifestation, followed by generalized cutaneous involvement. The absence of mucosal lesions and the restriction to the scalp contributed to a considerable delay in diagnosis and led to initial misdiagnoses, including fungal infections, neoplastic processes, and parasitic dermatoses.

The pivotal role of dermoscopy is highlighted in revealing specific vascular and surface features suggestive of an inflammatory or autoimmune etiology. Histopathological analysis showing suprabasal acantholysis, combined with direct immunofluorescence confirming intercellular IgG and C3 deposits in a net-like pattern, was instrumental in reaching the definitive diagnosis.

As emphasized, scalp involvement in pemphigus presents unique diagnostic challenges due to its rarity and the potential to mimic other dermatological conditions, which may delay appropriate management. Their review reinforces the necessity for clinicians to maintain a high index of suspicion for autoimmune blistering diseases such as PV even in uncommon locations or atypical morphologies.

Early consideration of pemphigus in the differential diagnosis of chronic, treatment-resistant scalp ulcers is essential to avoid misdiagnosis and unnecessary treatments, facilitating the timely initiation of immunosuppressive therapy. Ultimately, this case contributes to the growing awareness of the heterogeneous presentations of PV and the critical importance of a comprehensive diagnostic workup in ambiguous cases.

## References

[1] Bystryn JC, Rudolph JL (2005). Pemphigus. Lancet.

[2] Didona D, Maglie R, Eming R, Hertl M (2019). Pemphigus: current and future therapeutic strategies. Front Immunol.

[3] Sar-Pomian M, Rudnicka L, Olszewska M (2018). The significance of scalp involvement in pemphigus: a literature review. Biomed Res Int.

[4] Iwata H, Ujiie H, Nishie W, Shimizu H (2018). Pemphigus: etiology and pathogenesis. Dermatology.

[5] Grover C, Arora P, Manchanda V (2010). Tinea capitis in the pediatric population: a study from North India. Indian J Dermatol Venereol Leprol.

[6] Bolognia JL, Schaffer JV, Cerroni L (2018). Dermatology. Dermatology. 4th ed. Elsevier.

[7] Bonifaz A, Cruz M, Fierro L, Ponce RM (2007). Cutaneous leishmaniasis. Clin Dermatol.

[8] Ahmed AR, Moy R (1982). Death in pemphigus. J Am Acad Dermatol.

[9] Moriarty B, Hay R, Morris-Jones R (2012). The diagnosis and management of tinea. BMJ.

[10] Altmann S, Chahine A, Casale J, Forbes J, Ferrer-Bruker S (2021). Cutaneous type pemphigus vulgaris of the scalp: a rare unilesional presentation. SKIN.

[11] Bosseila M, Nabarawy EA, Latif MA, Doss S, ElKalioby M, Saleh MA (2019). Scalp pemphigus vulgaris mimicking folliculitis decalvans: a case report. Dermatol Pract Concept.

[12] Vega-Diez D, Lario AR, Zubiaur AG, Medina S (2021). Pemphigus vulgaris localized to the scalp with complete response to topical steroids. Int J Trichology.

[13] Alam M, Ratner D (2001). Cutaneous squamous-cell carcinoma. N Engl J Med.

[14] Hammers CM, Stanley JR (2016). Mechanisms of disease: pemphigus and bullous pemphigoid. Annu Rev Pathol.

[15] Kershenovich R, Hodak E, Mimouni D (2014). Diagnosis and classification of pemphigus and bullous pemphigoid. Autoimmun Rev.

[16] Tosti A, Torres F (2009). Dermoscopy in the diagnosis of hair and scalp disorders. Actas Dermosifiliogr.

[17] Porro AM, Seque CA, Ferreira MC, Enokihara MM (2019). Pemphigus vulgaris. An Bras Dermatol.

[18] Amagai M, Klaus-Kovtun V, Stanley JR (1991). Autoantibodies against a novel epithelial cadherin in pemphigus vulgaris, a disease of cell adhesion. Cell.

